# Sleep disturbance mediates the link between childhood trauma and clinical outcome in severe mental disorders

**DOI:** 10.1017/S0033291720000914

**Published:** 2021-10

**Authors:** Jannicke Fjæra Laskemoen, Monica Aas, Anja Vaskinn, Akiah Ottesen Berg, Synve Hoffart Lunding, Elizabeth Ann Barrett, Ingrid Melle, Carmen Simonsen

**Affiliations:** 1 NORMENT, Division of Mental Health and Addiction, Oslo University Hospital & Institute of Clinical Medicine, University of Oslo, Oslo, Norway; 2 Division of Mental Health and Addiction, Early Intervention in Psychosis Advisory Unit for South East Norway, Oslo University Hospital, Oslo, Norway

**Keywords:** Bipolar disorders, childhood trauma, psychosis, schizophrenia, sleep disturbances

## Abstract

**Background:**

The experience of childhood trauma is linked to more severe symptoms and poorer functioning in severe mental disorders; however, the mechanisms behind this are poorly understood. We investigate the relationship between childhood trauma and sleep disturbances in severe mental disorders including the role of sleep disturbances in mediating the relationship between childhood trauma and the severity of clinical symptoms and poorer functioning.

**Methods:**

In total, 766 participants with schizophrenia-spectrum (*n* = 418) or bipolar disorders (*n* = 348) were assessed with the Childhood Trauma Questionnaire. Sleep disturbances were assessed through the sleep items in the self-reported Inventory of Depressive Symptoms. Clinical symptoms and functioning were assessed with The Positive and Negative Syndrome Scale and the Global Assessment of Functioning Scale. Mediation analyses using ordinary least squares regression were conducted to test if sleep disturbances mediated the relationship between childhood trauma and the severity of clinical symptoms and poorer functioning.

**Results:**

Symptoms of insomnia, but not hypersomnia or delayed sleep phase, were significantly more frequent in participants with childhood trauma experiences compared to those without. Physical abuse, emotional abuse, and emotional neglect were significantly associated with insomnia symptoms. Insomnia symptoms partly mediate the relationship between childhood trauma and the severity of positive and depressive/anxiety symptoms, in addition to poorer functioning.

**Conclusion:**

We found frequent co-occurrence of childhood trauma history and current insomnia in severe mental disorders. Insomnia partly mediated the relationship between childhood trauma and the severity of clinical symptoms and functional impairment.

## Introduction

Childhood trauma is a well-documented risk factor for the development of severe mental disorders (Varese et al., 2012). In addition, individuals with severe mental illness that have experienced childhood trauma have more severe clinical symptoms than those without childhood trauma experiences (van Nierop et al., 2014). Sleep disturbances, such as subjective experiences of difficulties falling asleep, frequent awakenings, shorter duration of sleep, restless sleep, daytime fatigue and especially nightmares and anxiety dreams, are frequent sequelae of trauma exposure both in the short and the long term (Lavie, 2001). Sleep disturbances are also associated with more severe clinical symptomatology in severe mental disorders including the schizophrenia (SCZ) and the bipolar spectrum (Laskemoen et al., 2019). Thus, sleep disturbance may be a potential mechanism in the pathway from childhood trauma to the severity of clinical symptoms and functional impairment.

Childhood trauma has downstream consequences that include an increased risk of developing a mixture of anxiety, affective, and psychotic symptoms cutting across severe mental disorders (van Nierop et al., 2014). The frequency of childhood trauma in severe mental disorders is high (Church, Andreassen, Lorentzen, Melle, & Aas, 2017; Larsson et al., 2013) and linked to a more severe illness course and outcome, including earlier illness onset, increased risk of suicide attempts, and increased substance misuse (Aas et al., 2016*b*; Etain et al., 2013; Mohammadzadeh, Azadi, King, Khosravani, & Sharifi Bastan, 2019). More severe depressive, manic, and psychotic symptoms are also found in people with bipolar disorder(BD) exposed to childhood trauma (Agnew-Blais & Danese, 2016; Etain et al., 2013), while more severe depressive symptoms are found in people with SCZ exposed to childhood trauma (Aas et al., 2016a; Kelly et al., 2016; Sahin et al., 2013). The timing, severity, and duration of childhood trauma exposure are suggested important factors; however, these are poorly studied (Morgan & Gayer-Anderson, 2016). Several studies link specific trauma sub-types to certain symptom dimensions (Bentall et al., 2014; Varese et al., 2012). Yet, no specific trauma-subtype seems to stand out above others. A recent review (Gibson, Alloy, & Ellman, 2016) suggests that the relationship between childhood trauma and psychosis persist irrespective of trauma subtype, and that the experience of childhood trauma itself is the most important factor influencing clinical outcome in severe mental disorders.

Exactly how childhood trauma impacts on later clinical symptoms is however less clear. One possibility is that it causes prolonged neurobiological alterations, rendering the individual susceptible to the development of different mental disorders later in life. The specific developmental trajectories by which this takes place is, however, not fully known (Agorastos, Pervanidou, Chrousos, & Baker, 2019). To date, the majority of research has focused on the alterations of the stress response system as the putative main trajectory into the development of a wide range of mental disorders. More specifically, exposures to high levels of stress during sensitive periods of development in early childhood may over- or under-sensitize the neuroendocrine stress response, thereby inducing developmental disturbances linked to severe mental disorders in adulthood (Meerlo, Sgoifo, & Suchecki, 2008). Other pathophysiological mechanisms that are closely related to the stress response system, including sleep disturbances and circadian dysfunction, are potential mechanisms linking childhood trauma and later clinical symptoms (Agorastos et al., 2019). Both acute and chronic stress affect brain areas regulating sleep, with the potential to cause immediate and enduring sleep disturbance (Duclos et al., 2019; Lavie, 2001; Ouellet, Beaulieu-Bonneau, & Morin, 2015). Indeed, exposure to childhood trauma is associated with several types of sleep disturbances in adulthood, including insomnia symptoms, nightmare-related distress, and sleep apnea (Brindle et al., 2018; Kajeepeta, Gelaye, Jackson, & Williams, 2015). Sleep disturbances may in turn contribute to maladaptive stress regulation; further increasing vulnerability to the development of severe mental disorders (Meerlo et al., 2008).

There is increased recognition of sleep disturbance being involved in the pathophysiology and psychopathology of psychosis (Yates, 2016). Studies have causally linked sleep restriction to the development of psychotic symptoms (Reeve, Emsley, Sheaves, & Freeman, 2018*a*). Also, sleep disturbances are prominent irrespective of illness phase, i.e. both before and during manic and psychotic episodes (Allison & Harvey, 2008; Harvey et al., 2015; Kamath, Virdi, & Winokur, 2015). Moreover, sleep disturbances are associated with more severe positive, negative, and depressive symptoms as well as more severe functional impairment across severe mental disorders (Laskemoen et al., 2019; Reeve, Sheaves, & Freeman, 2015, 2018*c*).

Despite the high prevalence of both childhood trauma and sleep disturbances in severe mental disorders, previous studies have not investigated common links to clinical symptoms of severe mental disorders, with the exception of one study of persons with euthymic phase BD (Aubert et al., 2016). This study found a significant bivariate association between childhood emotional abuse and poorer current sleep quality; however, the association was no longer statistically significant after adjustment for clinical symptoms (suicidal behavior, anxiety, and the use of anxiolytic medication). Another study examined the joint influences of sleep disturbance and trauma on psychotic-like experiences in a sample of otherwise healthy undergraduate students (Andorko et al., 2018), and found that both previous trauma exposure and sleep disruptions predicted psychotic-like experiences. The effect of trauma exposure did, however, not reach the level of statistical significance after correction for sleep disturbances in multivariate analyses. These studies illustrate that the relationship between childhood trauma and sleep disturbance may be confounded by other factors. Age, gender, diagnostic group (SCZ or BD), recent alcohol or drug use, a history of alcohol or drug dependency, medication with sedative effects, or weight are factors previously shown to be related to sleep disturbances, and may also be influenced by childhood trauma. To what extent sleep disturbances mediate the association between exposure to childhood trauma and the severity of clinical symptoms and functional impairment in psychotic disorders has, to best of our knowledge, never been investigated.

### Aims of the study

We have previously shown that the frequency of sleep disturbance is high across severe mental disorders (SCZ and BD), and that sleep disturbances are associated with more severe clinical symptoms and functional impairment (Laskemoen et al., 2019). We here investigate the relationship between sleep disturbances (any sleep disturbance and sleep disturbance subtypes) and childhood trauma (any childhood trauma and childhood trauma subtypes). Based on the previous findings of links between sleep disturbances and clinical symptoms/poorer functioning, and between childhood traumas and clinical symptoms/poorer functioning, we examine a theoretical mediation model to test if the relationship between childhood trauma and clinical symptoms/poorer functioning is mediated by sleep disturbances. We also evaluate if this theoretical mediation model is confounded by age, gender, diagnostic group (SCZ or BD), recent alcohol or drug use, a history of alcohol or drug dependency, medication with sedative effects, or weight (BMI).

## Materials and methods

### Participants

The current study is part of the Thematically Organized Psychosis (TOP) Research Study at the Norwegian Centre for Mental Disorders Research (NORMENT) in Oslo, Norway. This study includes 766 participants with psychotic disorders (SCZ = 418, BD = 348), recruited between 2003 and 2019. In the SCZ group, 223 participants had an SCZ diagnosis, 61 had a schizoaffective disorder, 30 had a schizophreniform disorder, and 104 had a diagnosis of other psychotic disorders. In the BD group, 223 participants had a diagnosis of bipolar I, 110 bipolar II, and 25 bipolar NOS. Participants went through a physical examination and interview about somatic illness history and current health status, completed by a medical doctor. Based on this examination and interview, in addition to consultation of journal notes, participants were excluded from the TOP study if they had a history of head injury needing hospitalization, neurological disorder, and from the current analyses if they had a known primary sleep disorder (based on self-report) such as restless legs syndrome or obstructive sleep apnea. Having IQ below 70 and age outside 18–65 years were also part of the general exclusion criteria, determined based on the information from neurocognitive assessments and clinical interviews. Participants with recent intake of drugs or alcohol, or with a history of drug or alcohol abuse or dependency, were not excluded to ensure a representative sample. Yet, all had to be abstinent (not visibly intoxicated, or affecting clinical presentation) at the time of interviews and testing. All participants gave written informed consent after being given thorough information about the study protocol and procedures, and the study was approved by ‘The Regional Committee for Research Ethics’ and ‘The Norwegian Data Inspectorate’.

### Demographics and clinical characteristics

For diagnostic evaluation, all participants were assessed with the Structured Clinical Interview for DSM-IV (SCID-IV) (First, Spitzer, Gibbon, & Williams, 1995). The Positive and Negative Syndrome Scale (PANSS) (Kay, Fiszbein, & Opler, 1987), applying Wallwork's five-factor model (Wallwork, Fortgang, Hashimoto, Weinberger, & Dickinson, 2012), was used to assess current symptom levels using the factors PANSS positive, PANSS negative, PANSS disorganized/concrete, PANSS excited, and PANSS depressed. As the PANSS depressed includes the items G2 Anxiety, G3 Guilt Feelings, and G6 Depression Posturing, we have in the current paper used the more precise label ‘PANSS depression/anxiety’ factor. Current illness phase was stratified into those with and without psychosis in participants with SCZ-spectrum disorders and those with and without depression and mania in those with BDs. Current psychosis or symptomatic remission was defined according to the internationally standardized criteria (Andreasen et al., 2005), in which remission requires scores below 4 on the following PANSS items: positive symptoms (P1-delusions, G9-unusual thought content, P3-hallucinations), disorganized symptoms (P2-conceptual disorganization, G5-mannerisms/posturing), and negative symptoms (N1-blunted effect, N4-social withdrawal, N6-lack of spontaneity). Consequently, those scoring 4 or above on any of these items were considered to be psychotic. Current depression was defined using Inventory of Depressive Symptoms – Clinician-rated scale (IDS-C) (Rush, Gullion, Basco, Jarrett, & Trivedi, 1996), with a total score equal to or above 14. We used the Young Mania Rating Scale (Young, Biggs, Ziegler, & Meyer, 1978) with a total score equal to or above eight to define current mania. Those that neither met criteria for current depression or mania were defined as currently euthymic. To assess the level of functioning, we used the functioning subscale of the split-version of the Global Assessment of Functioning Scale (GAF-F) (Pedersen & Karterud, 2012). Clinical interviews and medical charts were used to gather information on recent use (past 2 weeks) of alcohol and illegal drugs (number of units for alcohol and number of times used for any relevant drug), as well as the current type(s) of use of psychotropic medication (antipsychotics, anxiolytics/hypnotics, antidepressants, antiepileptics, and/or lithium). All psychotropic medication that had sedation marked as a main or major side-effect in The Norwegian Pharmaceutical Product Compendium (Felleskatalogen, 2018) were classified as medication with sedative effects. To further enhance this dichotomized classification of psychotropic medication having sedative effects/not, we evaluated their known modes of action on neurotransmitters involved in promoting sleepiness (histaminergic/muscarinergic) such as e.g. quetiapine and olanzapine.

### Childhood trauma

To assess traumatic experiences in childhood, we applied the Norwegian version of the Childhood Trauma Questionnaire-Short Form (CTQ-SF) (Bernstein et al., 2003). This self-report questionnaire comprises 28 items rated on a five-point Likert scale ranging from 1 (never true), 2 (rarely true), 3 (sometimes true), 4 (often true) to 5 (very often true) forming five subscales; emotional abuse (EA), physical abuse (PA), sexual abuse (SA), physical neglect (PN), and emotional neglect (EN) in addition to the total trauma score. We used these childhood trauma data both as a continuous variable and as variables dichotomized into ‘no trauma exposure’ (none or mild exposure) *v.* ‘trauma exposure’ (moderate or severe exposure), based on the cut-off scores recommended by Bernstein and Fink (1998).

### Sleep disturbances

Rating of sleep disturbances was based on the four first items from the IDS-C (Rush et al., 1996); difficulty falling asleep (item 1; Sleep Onset Insomnia), difficulty maintaining sleep (item 2; Mid-Nocturnal Insomnia), early awakening (item 3; Early Morning Insomnia), and hypersomnia (item 4; Hypersomnia). The items are rated on a four-point Likert scale (0–3) in which higher scores represent more subjective disturbance based on sleep experience over the past 7 days. IDS-C has been used in several prior studies to differentiate between – and conceptualize – sleep disturbances (Laskemoen et al., 2019; Steinan et al., 2016*a*, 2016*b*). These four sleep items have also been validated as the measures of insomnia and hypersomnia, and have been shown to adequately predict a clinical diagnosis of sleep disorders (Gruber et al., 2009; Kaplan, Gruber, Eidelman, Talbot, & Harvey, 2011; Soehner, Kaplan, & Harvey, 2014; Sylvia et al., 2012). In line with previous studies (Laskemoen et al., 2019; Steinan et al., 2016*a*, 2016*b*), we define the following sleep disturbances based on the IDS-C (reminding that these definitions represent current symptoms of sleep disturbances and are not diagnostic categories).

1. **Insomnia** corresponds to: (a) a score of ⩾ 2 on *Sleep Onset Insomnia* (more than half the time it takes minimum 30 min to fall asleep); (b) a score of = 3 on *Mid-Nocturnal Insomnia* (more than half the time, waking up happens more than once per night and one stays awake for 20 min or more); or (c) a score of ⩾ 1 on *Early Morning Insomnia* (more than half the time, one wakes up more than 30 min before needing to get up). In addition, a score of = 0 on the *Hypersomnia* item (sleeps no more than 7–8 h a night, without naps) was a prerequisite.
2. **Insomnia total score** corresponds to the sum of item (1) *Sleep Onset Insomnia*, item (2) *Mid-Nocturnal Insomnia*, and item (3) *Early Morning Insomnia.*
3. **Hypersomnia** corresponds to a score of ⩾1 on the *Hypersomnia* item (sleeping up to 10 h per day) with no evidence of insomnia.
4. **Delayed sleep phase (DSP)** corresponds to a score of ⩾3 on *Sleep Onset Insomnia* (more than half the time, it takes more than 60 min to fall asleep), and a score of ⩾1 on the *Hypersomnia* item.
5. **Any sleep disturbance** corresponds to a score over cut-off on any of the sleep disturbances described**.**

All of the above conceptualizations of sleep disturbances are dichotomized variables with the exception of insomnia total score which can be measured continuously.

### Statistical analyses

Statistical analyses were performed using The Statistical Package for the Social Sciences (SPSS INC, Chicago, IL, USA, version 25). We first examined the frequency of the different sleep disturbances (any sleep disturbance, insomnia, hypersomnia, and delayed sleep phase; dichotomous variables) amongst participants with and without childhood trauma (dichotomous variables) using χ^2^ statistics. We then conducted follow-up analyses, repeating the same analyses separately for the SCZ and BD groups. Secondly, the differences in the reported magnitude of childhood trauma subtypes (assessed as continuous variables) between those with and without the different sleep disturbances (insomnia, hypersomnia, and delayed sleep phase; dichotomous variables) were examined with Mann–Whitney *U* tests due to skewed distributions of childhood trauma data. Follow-up analyses were conducted repeating the same analyses separately for the SCZ and BD groups.

**Fig. 1.**
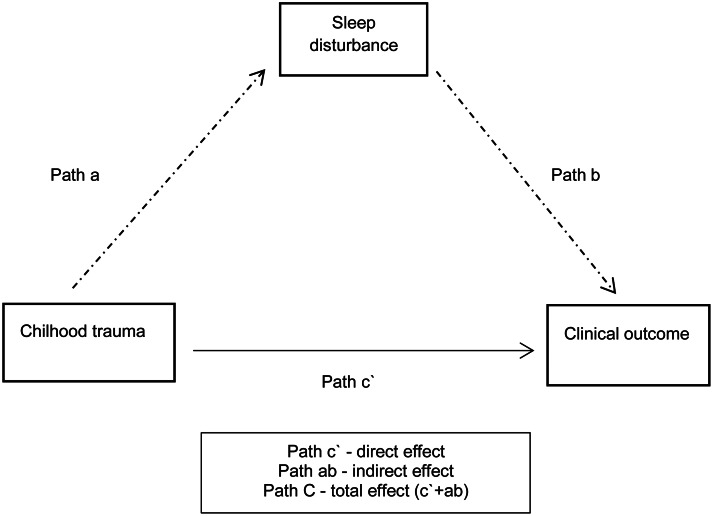
Overall model.

For our third research aim, we examined whether the presence of a sleep disturbance mediated the relationship between childhood trauma total score and clinical outcome, using the following approach to select variables for the model (see Fig. 1). We first examined *path c*, calculating *Spearman's correlations* between the predictor variable (childhood trauma total) and the clinical outcome-dependent variables (PANSS positive, negative, disorganized, excited, depressive/anxiety, and GAF-F). Childhood trauma total was chosen as a predictor variable for several reasons. The relationship between childhood trauma and clinical outcome has been shown to persist regardless of trauma type. Furthermore, the amount of trauma exposure is suggested to be important (Gibson et al., 2016). Lastly, partitioning out a specific trauma sub-type would mean losing statistical power. Secondly, focusing on *path b*, correlations between the mediator (sleep disturbance) and the clinical outcome-dependent variables were calculated. Clinical outcome measures that were significantly correlated with both the predictor (childhood trauma total) and the mediator (sleep disturbance) at a Bonferroni-adjusted *p* level (0.05/6 clinical outcome measures = new *p* value 0.008) were selected as dependent (outcome) variables for the mediation model.

Based on findings from our second aim, the sleep disturbance(s) found to be significantly associated with the predictor variable (childhood trauma total) would then serve as a mediator in the model. We conducted mediation analyses based on *regression analyses*; applying Haye's PROCESS tool version 3.3 for SPSS. We used model 4 designs, in which total childhood trauma score was set as a predictor, sleep disturbance was set as a mediator, and the different clinical outcome variables were set as dependent variables, respectively. We examined *path a* (from the predictor to the mediator), *path b* (from the mediator to the dependent variable), *path c’* (the direct effect of the predictor to the dependent variable) and *path C* (the total effect of the predictor on the dependent variable) as well as the bootstrapped indirect effect and its confidence interval, using 5000 bootstrap samples. We calculated the proportion of the total effect mediated (in percentage) as the ratio of the indirect effect to the total effect, as well as the partially standardized indirect effects.

Lastly, follow-up analyses were conducted including covariates significantly associated with either the predictor variable (childhood trauma total) or the sleep disturbance(s) found to be significantly associated with the predictor variable (childhood trauma total).

## Results

### Demographics and clinical characteristics

An overview of demographic and clinical characteristics for the total sample is shown in Table 1. As seen under *clinical variables* and *current illness phase*, the majority of participants were currently symptomatic.

**Table 1.** Demographics and clinical characteristics in the total sample

	Total sample *N* = 766	Childhood trauma	No childhood trauma
Demographics			
Age, mean ± s.d.	30.9 ± 10.6	31.6 (10.6)	30.1 (10.6)
Gender, male *n* (%)	384 (50.1)	178 (48.8)	192 (52.2)
Education in years, mean ± s.d.	13.9 ± 3.1	13.5 (3.2)	14.3 (2.9)
Childhood trauma *n* (%)^a^	365 (49.8)		
Physical abuse *n* (%)^b^	92 (12.1)		
Sexual abuse *n* (%)^c^	122 (16.3)		
Emotional abuse *n* (%)^d^	208 (27.7)		
Emotional neglect *n* (%)^e^	209 (28.0)		
Physical neglect *n* (%)^f^	167 (22.1)		
Clinical variables			
Insomnia *n* (%)	344 (44.9)	188 (51.5)	143 (38.9)
Hypersomnia *n* (%)	231 (30.2)	96 (26.3)	125 (34.0)
Delayed sleep phase *n* (%)	58 (7.6)	27 (7.4)	30 (8.2)
Any sleep disturbance *n* (%)	575 (75.1)	284 (77.8)	268 (72.8)
Alcohol units last two weeks, mean ± s.d.	6.5 ± 12.8	6.9 (14.0)	6.3 (11.4)
Drug use last two weeks, mean ± s.d.	0.4 ± 2.0	0.5 (2.3)	0.3 (1.8)
Lifetime alcohol dependency, yes (%)	97 (12.7)	57(15.6)	37 (10.1)
Lifetime drug dependency, yes (%)	128 (16.7)	72 (19.7)	52 (14.1)
PANSS positive, mean ± s.d.	8.0 ± 4.0	8.5 (4.2)	7.5 (3.8)
PANSS negative, mean ± s.d.	11.1 ± 5.3	11.0 (4.9)	11.1 (5.6)
PANSS disorganized, mean ± s.d.	6.3 ± 2.6	6.4 (2.7)	6.2 (2.5)
PANSS excited, mean ± s.d.	5.5 ± 1.9	5.6 (2.0)	5.3 (1.8)
PANSS depression/anxiety, mean ± s.d.	7.9 ± 3.0	8.6 (3.1)	7.3 (2.8)
GAF-F, mean ± s.d.	50.1 ± 13.9	47.5 (12.5)	52.6 (14.6)
IDS-C total, mean ± s.d.^g^	17.4 (11.3)	20.1 (11.5)	15.1 (10.7)
YMRS total, mean ± s.d.^h^	3.7 (4.5)	4.4 (4.7)	3.1(4.4)
*Current illness phase*^i^			
Schizophrenia-spectrum disorders, *n* = 418			
Psychotic *n* (%)	297 (72.1)	157 (76.2)	126 (67.0)
Remission *n* (%)	115 (27.9)	49 (23.8)	62 (33.0)
Bipolar disorders, *n* = 348:			
Depressed *n* (%)	189 (55.4)	95 (62.1)	87 (49.7)
Manic *n* (%)	50 (14.4)	29 (18.6)	20 (11.3)
Euthymic *n* (%)	131 (38.5)	46 (30.1)	79 (45.4)
Medication and somatic variables			
Antipsychotics *n* (%)	523 (68.3)	237 (64.9)	262 (71.2)
Mood stabilizers *n* (%)	219 (28.6)	107 (29.3)	104 (28.3)
Antidepressants *n* (%)	228 (29.8)	117 (32.1)	96 (26.1)
Anxiolytics/hypnotics *n* (%)	75 (9.8)	44 (12.1)	28 (7.6)
Medication with sedative effects *n* (%)	354 (46.2)	169 (46.3)	169 (45.9)

PANSS, Positive and Negative Syndrome Scale. PANSS was organized after Wallwork's five-factor model; GAF-F, Global Assessment of Functioning scale; BMI, body mass index. Mood stabilizer refers to lithium or antiepileptics.

a 95.7% (*n* = 733) of the participants had data on childhood trauma.

b 99.1% (*n* = 759) of the participants had data on physical abuse.

c 97.5% (*n* = 747) of the participants had data on sexual abuse.

d 98.0% (*n* = 751) of the participants had data on emotional abuse.

e 97.4% (*n* = 746) of the participants had data on emotional neglect.

f 98.7 (*n* = 756) of the participants had data on physical neglect.

g = 95.2 (*n* = 729) of the participants had data on IDS-C total.

h = 99.5 (*n* = 762) of the participants had data on YMRS total.

i = Current illness phase is based on cut-off scores from PANSS items, IDS-C, and YMRS.

### The frequency of sleep disturbance in those with and without childhood trauma

As illustrated in Table 1, 50% of the total sample had experienced childhood trauma. The frequency of *any sleep disturbance* was not significantly different in those with childhood trauma experiences (78%) and those without (73%) (χ^2^ = 2.45, *p* = 0.118). However, the frequency of insomnia was significantly *higher* in those with childhood trauma experiences (52%) *v.* those without (39%) (χ^2^ = 11.84, *p* = 0.001), with 26% of the total sample reporting the occurrence of both childhood trauma experiences and insomnia. In contrast, the frequency of hypersomnia was significantly *lower* in those with childhood trauma experiences (26%) compared to those without (34%) (χ^2^ = 5.11, *p* < 0.05). The frequency of DSP did not differ significantly between participants with (7%) and without childhood trauma experiences (8%) (χ^2^ = 0.15, *p* = 0.703). Follow-up analyses showed that for both SCZ and BD, insomnia was the only sleep disturbance that was significantly higher in those with childhood trauma experiences compared to those without (SCZ: 52% *v.* 39%, χ^2^ = 6.87, *p* < 0.01; BD: 49% *v.* 38%, χ^2^ = 4.66, *p* < 0.05).

### The magnitude of childhood trauma subtypes and different sleep disturbances

As illustrated in Table 2, the level of *childhood trauma total*, *physical abuse*, *emotional abuse*, and *emotional neglect* were significantly higher in participants with insomnia compared to those without; while significantly lower in those with hypersomnia compared to those without. The level of s*exual abuse* and *physical neglect* were not significantly different between participants with or without insomnia or hypersomnia. There was no difference between participants with and without DSP for childhood trauma total or any of the childhood trauma subtypes.

**Table 2.** The relationship between childhood trauma subtypes and different sleep disturbances in severe mental disorders (Mann–Whitney *U* test)

	Insomnia			Hypersomnia			Delayed sleep phase		
	Median Insomnia *N* = 344	Median no Insomnia *N* = 422	Statistics	Median Hypersomnia *N* = 231	Median no Hypersomnia *N* = 535	Statistics	Median DSP *N* = 58	Median no DSP *N* = 708	Statistics
Childhood trauma total	40.0	38.0	*U* = 52 813.50 *z* = −3.39 *p* = 0.001	38.0	40.0	*U* = 46 063.50 *z* = −2.72 *p* < 0.01	38.0	39.0	*U* = 17 355.00 *z* = −0.00 *p* = 0.999
Physical abuse	5.0	5.0	*U* = 62 990.00 *z* = −3.18 *p* < 0.001	5.0	5.0	*U* = 55 018.00 *z* = −2.29 *p* < 0.05	5.0	5.0	*U* = 19 704.00 *z* = −0.45 *p* = 0.655
Sexual abuse	5.0	5.0	*U* = 64 928.00 *z* = −1.8 *p* = 0.072	5.0	5.0	*U* = 56 587.00 *z* = −1.01 *p* = .312	5.0	5.0	*U* = 19 483.00 *z* = −0.15 *p* = 0.882
Emotional abuse	10.0	9.0	*U* = 57 187.50 *z* = −4.26 *p* < 0.001	8.0	10.0	*U* = 50 591.50 *z* = −3.32 *p* < 0.001	10.0	9.0	*U* = 18 756.00 *z* = −0.65 *p* = 0.514
Emotional neglect	11.0	10.0	*U* = 61 418.00 *z* = −2.53 *p* < 0.01	10.0	11.0	*U* = 53 263.00 *z* = −1.99 *p* < 0.05	11.0	11.0	*U* = 18 794.00 *z* = −0.14 *p* = 0.890
Physical neglect	7.0	6.0	*U* = 65 805.00 *z* = −1.66 *p* = 0.098	6.0	7.0	*U* = 56 890.50 *z* = −1.23 *p* = .221	7.0	7.0	*U* = 19 654.50 *z* = −0.38 *p* = 0.707

Follow-up analyses showed that in the SCZ group; *childhood trauma total*, *physical abuse*, *emotional abuse*, and *emotional neglect* were significantly higher in participants with insomnia compared to those without; whilst *childhood trauma total*, *physical abuse*, and *emotional abuse* were significantly lower in those with hypersomnia compared to those without. In the BD group, only *emotional abuse* was significantly higher in participants with insomnia compared to those without, whilst significantly lower in those with hypersomnia compared to those without.

This shows that insomnia is common in participants with childhood trauma, and in the context of *physical abuse*, *emotional abuse*, and *emotional neglect*. Insomnia was therefore chosen as the mediator in the analyses of the relationship between childhood trauma and clinical outcome, used as a continuous variable labeled ‘*insomnia total*’. The relationship between the predictor (childhood trauma total) and the mediator (insomnia total) (path a) was re-stated in a significant bivariate correlation (Spearman's *ρ* = 0.196, *p* < 0.001).

### Testing the theoretical model; sleep disturbance as a mediator between childhood trauma and clinical outcome

#### Relationship between insomnia total, childhood trauma total, and clinical outcome

Both *Childhood trauma total* and *Insomnia total* were significantly associated (using a threshold of 0.008) with PANSS positive and PANSS depressive/anxiety, as well as GAF-F (Table 3). Consequently, further mediation analyses were conducted separately for PANSS positive, PANSS depressione/anxiety, and functioning GAF F.

**Table 3.** The relationship between sleep disturbance, childhood trauma, and clinical outcome in severe mental disorders (Spearman*’*s correlations)

	PANSS Positive	PANSS Negative	PANSS Disorganized/concrete	PANSS Excited	PANSS Depression/anxiety	GAF-F
Childhood trauma total	*r* = .142 *p* < 0.001	*r* = −0.003 *p* = 0.934	*r* = 0.019 *p* = 0.620	*r* = 0.097 *p* = 0.01	*r* = 0.256 *p* < 0.001	*r* = −0.180 *p* < 0.001
Insomnia total	*r* = .216 *p* < 0.001	*r* = .114 *p* < 0.01	*r* = 0.068 *p* = 0.062	*r* = 0.105 *p* < 0.01	*r* = 0.339 *p* < 0.001	*r* = −0.158 *p* < 0.001

GAF, Global Assessment of Functioning scale; PANSS, Positive and Negative Syndrome Scale. PANSS was organized after Wallwork's five-factor model.

#### Insomnia total as a mediator in the relationship between childhood trauma and clinical outcome

Three separate mediation analyses were conducted to evaluate whether the effect of childhood trauma total on PANSS positive, PANSS depression/anxiety, and GAF-F, respectively, was mediated by insomnia total. As none of the confidence intervals contain zero, there is a statistically significant indirect effect of childhood trauma total via insomnia total for all three clinical outcome measures (Table 4). Insomnia mediated 25% of the effect of childhood trauma on PANSS positive, 26% of the effect on PANSS depression/anxiety, as well as 12% of the effect on GAF-F.

**Table 4.** Insomnia as a mediator in the relationship between childhood trauma and clinical outcome (total effect, direct effect, and indirect effect)

	Total effect, estimate (s.e.), *p* value	Direct effect, estimate (s.e.), *p* value	Indirect effect, bootstrap confidence interval	Partially standardized indirect effect	Proportion mediated % (ab/C)
PANSS positive	0.9684, (0.2925) *p* = 0.001	0.7228, (0.2904) *p* = 0.0130	0.1158–0.4039	0.0613	25%
PANSS depression/anxiety	1.2307, (0.2188) *p* < 0.001	0.9139, (0.2085) *p* < 0.001	0.1614–0.4896	0.1042	25.7%
GAF-F	−5.1711, (0.9948) *p* < 0.001	−4.5423, (0.9948) *p* < 0.001	−1.0996 to −0.2563	−0.0455	12%

Spearman's correlations, *t* test, or Mann–Whitney *U* tests were conducted to evaluate the bivariate associations to covariates [age, gender, diagnostic group (SCZ or BD), recent alcohol or drug use, a history of alcohol or drug dependency, medication with sedative effects, or weight (BMI)], data not shown. Lower age was significantly correlated with insomnia total (*r* = −0.071, *p* < 0.05) and higher age with childhood trauma total (*r* = 0.117, *p* < 0.005). Higher recent intake of drugs was significantly associated with higher insomnia total score (*r* = 0.120, *p* < 0.01), whilst having a history of drug dependency was significantly associated with higher childhood trauma total (*U* = 32 042.5, *p* < 0.05). Consequently, age, recent intake of drugs, and history of drug dependency were included as covariates in the three separate follow-up mediation analyses. After adjusting for these covariates, the indirect effect of childhood trauma total via insomnia total for all three clinical outcome measures remained statistically significant from zero.

## Discussion

This is the first study on severe mental disorders to show that people with a history of childhood trauma more often experience symptoms of insomnia than those without a history of childhood trauma, and that insomnia is a significant mediator of the effect of childhood trauma on the severity of positive symptoms, symptoms of depression/anxiety, and poorer functioning.

Whilst insomnia is significantly more frequent in participants with childhood trauma, the opposite is the case for hypersomnia. These differences in associations could be based on different biological underpinnings of the two sleep disturbances. Insomnia is characterized by physiological, cognitive, and affective hyperarousal (Levenson, Kay, & Buysse, 2015), a theoretically plausible consequence of trauma exposure. The disinhibition of sleep-induced transmission and reduced activity in wake-promoting neurons that may be seen in hypersomnia (Carbonell & Leschziner, 2017) is possibly less likely to be a consequence of trauma-related stress response. Consequently, childhood trauma and hypersomnia might not share common underlying mechanisms; indeed, a state of hyper-arousal would prohibit hypersomnia.

Although the relationship between childhood trauma and sleep disturbance primarily has been investigated in the general population, our finding of an association between total childhood trauma and insomnia fit well with a review of retrospective cohort studies (Brindle et al., 2018). Also, in a recent study of young individuals with a history of depression (Hamilton, Brindle, Alloy, & Liu, 2018), childhood emotional neglect was found to predict insomnia symptoms. This is in line with our findings that three subtypes of childhood trauma (*physical abuse*, *emotional abuse*, and *emotional neglect*) were significantly associated with insomnia in the total sample, most likely driven by the associations in the SCZ group. For BD, we mainly found associations to emotional abuse. We have identified one other study of severe mental disorders in this area (Aubert et al., 2016), showing a significant association between *emotional abuse* and poorer sleep quality in euthymic persons with BD. In the latter study, the authors decided to adjust for clinical aspects, including anxiety and use of anxiolytic medication as potential confounders, i.e. not considering the possibility that sleep disturbances could mediate the association between trauma and clinical symptoms. Taken together, this supports the idea that *emotional abuse* might be an especially salient trauma subtype in BD.

The existing studies of sleep and trauma in clinical samples (Aubert et al., 2016; Hamilton et al., 2018), including the current, have not found any association between sexual abuse and insomnia. This is in contrast to the studies of non-clinical samples (Kajeepeta et al., 2015; Lind, Aggen, Kendler, York, & Amstadter, 2016; Steine et al., 2012). Our data have no indications of why. However, studies suggest distinct trauma subtypes differentially affect biological stress responses (Kuhlman, Geiss, Vargas, & Lopez-Duran, 2015), and thus may interact with underlying vulnerabilities in different ways. In this vein, different trauma subtypes may also affect sleep disturbances differently.

Our finding that insomnia partially mediates the relationship between childhood trauma and the severity of positive symptoms, depression/anxiety symptoms, and dysfunction lends support to our proposed theoretical model. The findings have implications for understanding how stress impacts on the severity of psychosis, and could imply that sleep disturbances following childhood trauma interact with the stress regulatory system further exacerbating clinical symptoms. This line of reasoning is supported by recent knowledge about the relations between activities of the stress response system and the circadian system (Neumann, Schmidt, Brockmann, & Oster, 2019). Dysregulations of both systems may progressively change the underlying properties of brain systems that regulate neuroendocrine function, and thus play an important role in the development of stress-related disorders, potentially creating a vicious circle. Indeed, sleep disturbances following trauma exposure have been suggested as a core pathway mediating the neurobiological consequences of this exposure (Landgraf, McCarthy, & Welsh, 2014; Lavie, 2001; Teicher et al., 2017). Furthermore, recent studies support that insomnia is a contributory factor in the development of psychosis (Freeman et al., 2017; Reeve et al., 2015, 2018*a*), and that the relationship between insomnia and psychotic experiences is mediated by negative affect, such as anxiety and depression (Reeve, Nickless, Sheaves, & Freeman, 2018*b*). Our findings thus suggest that insomnia in persons exposed to childhood trauma could be one of the driving forces behind worsening of clinical symptoms and functional impairment in severe mental disorders.

In addition to these theoretical aspects, the current study has several clinical implications. Firstly, it identifies a large subgroup of patients with co-occurring childhood trauma and current insomnia, and advocates routine assessment of both childhood trauma and sleep disturbances in clinical practice. Moreover, the suggestion that insomnia could be causally related to the severity of clinical symptoms and functional impairment in this subgroup supports the importance of treating insomnia to improve psychotic symptoms (Freeman et al., 2017). Despite growing evidence that cognitive-behavioral therapy for insomnia is feasible, acceptable, and effective also in psychosis (Freeman et al., 2017; Waite et al., 2016), formal assessment and recommended interventions for sleep disturbances are rarely used in clinical practice (Rehman et al., 2017).

Some methodological limitations should be addressed: Our data are cross-sectional, limiting our possibility to infer on causal associations. Sleep disturbances are based on items from a rating scale primarily designed to assess depressive symptoms (IDS-C). The four sleep items applied have nevertheless been found valuable in the prediction of diagnoses of sleep disorders (Gruber et al., 2009; Kaplan et al., 2011; Soehner et al., 2014; Sylvia et al., 2012) and have been used in previous studies (Laskemoen et al., 2019; Steinan et al., 2016*a*, 2016*b*). As night-mares are common symptoms of trauma, and might cause frequent wake-ups that may lead to high insomnia scores, night-mare disorders should ideally have been assessed. Our measure of childhood traumas is a questionnaire based on retrospective trauma experience, thus recall bias may have influenced the reports. However, evidence of systematic recall bias in the reports of retrospective trauma has not been found in previous studies designed to detect this phenomenon (Edwards et al., 2001).

## Conclusion and future directions

This is the first study to identify a large sub-group (26%) of people with severe mental disorders having both childhood trauma experiences and current insomnia symptoms. Importantly, this study provides evidence of a theoretically based mediation model, showing that insomnia partly mediates the association between childhood trauma and the severity of clinical symptoms and functional impairment across severe mental disorders. Longitudinal studies are now needed to disentangle the relationship between childhood trauma, sleep disturbance, and clinical outcome, employing both subjective and objective measures of sleep disturbances. As the current study may suggest a causal trajectory from childhood trauma experiences via insomnia to the severity of clinical symptoms, future studies should also investigate whether the combination of childhood trauma experience and insomnia might represent cumulative risk factors for the development of severe mental disorders. Insomnia could thus be a treatment target in at-risk individuals.
